# Protein dynamics developments for the large scale and cryoEM: case study of *ProDy* 2.0

**DOI:** 10.1107/S2059798322001966

**Published:** 2022-03-16

**Authors:** James Michael Krieger, Carlos Oscar S. Sorzano, Jose Maria Carazo, Ivet Bahar

**Affiliations:** a Biocomputing Unit, Centro Nacional de Biotecnología (CSIC), Calle Darwin 3, 28049 Madrid, Spain; b Department of Computational and Systems Biology, School of Medicine, University of Pittsburgh, 800 Murdoch Building, 3420 Forbes Avenue, Pittsburgh, PA 15213, USA

**Keywords:** computational biophysics, signature protein dynamics, elastic network model, normal mode analysis, ensemble essential dynamics, principal component analysis, cryo-electron microscopy, pseudoatoms

## Abstract

Cryo-electron microscopy (cryoEM) has become a well established technique with the potential to produce structures of large and dynamic supramolecular complexes that are not amenable to traditional approaches for studying structure and dynamics. The size and low resolution of such molecular systems often make structural modelling and molecular dynamics simulations challenging and computationally expensive. This, together with the growing wealth of structural data arising from cryoEM and other structural biology methods, has driven a trend in the computational biophysics community towards the development of new pipelines for analysing global dynamics using coarse-grained models and methods. At the centre of this trend has been a return to elastic network models, normal mode analysis (NMA) and ensemble analyses such as principal component analysis, and the growth of hybrid simulation methodologies that make use of them. Here, this field is reviewed with a focus on *ProDy*, the Python application programming interface for protein dynamics, which has been developed over the last decade. Two key developments in this area are highlighted: (i) ensemble NMA towards extracting and comparing the signature dynamics of homologous structures, aided by the recent *SignDy* pipeline, and (ii) pseudoatom fitting for more efficient global dynamics analyses of large and low-resolution supramolecular assemblies from cryoEM, revisited in the *CryoDy* pipeline. It is believed that such a renewal and extension of old models and methods in new pipelines will be critical for driving the field forward into the next cryoEM revolution.

## Abstract 2

New computational biophysics pipelines for analysing the global dynamics of structural ensembles and large, dynamic complexes resolved by cryoEM are reviewed.

## 1. Introduction

With the increasing popularity of cryo-electron microscopy (cryoEM) for structural studies of biological macromolecules following the resolution revolution, it is becoming increasingly clear that single structural snapshots are insufficient to explain molecular mechanisms of action. Instead, there has been a growing awareness that proteins, like all molecules, are intrinsically dynamic. They undergo various changes in structure as a result of thermal fluctuations and interactions with other molecules, enabling them to visit many conformational states under most conditions, often involving global rearrangements of the whole structure. The fact that this takes place under the near-physiological conditions captured by plunge-freezing, even when they are trapped in biochemically controlled functional states, has led to a growing interest in understanding their inherent heterogeneity and dynamics.

The growing data from structural biology, including cryoEM, have provided great support for the hypothesis of ‘structure-encoded’ global motions constrained by the internal interaction network of protein complexes that are critically important for carrying out biological functions and subject to optimization during evolution (Zhang *et al.*, 2020 ▸). This has resulted in the renewal of techniques focusing on global dynamics (Bahar *et al.*, 2010 ▸, 2017 ▸), with fast analytical matrix-decomposition methods, such as normal mode analysis (NMA) and principal component analysis (PCA), being especially popular. Their elegance and efficiency lies in their ability to derive the dynamic covariance of atom positions from nothing more than the interaction forces inherent to a single structure for NMA or a small number of alternative positions in related structures for PCA, and to simply decompose the resulting covariance into a set of modes of motion as described below. These techniques very readily lend themselves to the use of coarse-grained (CG) representations, such as residue-resolution elastic network models (ENMs), given their robustness to the functional form of the interaction potential and the level of coarse-graining (Doruker *et al.*, 2000 ▸, 2002 ▸; Hinsen, 1998 ▸; Tirion, 1996 ▸). These together enable much more efficient evaluations of global motions than the traditionally popular all-atom molecular dynamics (MD) simulations (Hollingsworth & Dror, 2018 ▸). Thus, the cryoEM revolution is driving an associated revolution in computational biophysics, with many groups developing new pipelines to handle the increasingly large numbers of related structures and large sizes of macromolecular complexes.

In parallel, there has been an explosion in the use of application programming interfaces (APIs) and servers that allow the pipelining of these methods, including the *ProDy* Python package (Zhang, Krieger, Zhang *et al.*, 2021 ▸) (with more than two million downloads since its inception in 2011; Bakan *et al.*, 2011 ▸) and the *DynOmics* webserver (Li *et al.*, 2017 ▸) from the Bahar laboratory, the *Bio*3*D* *R* packages and *Bio*3*D-web* (Grant *et al.*, 2021 ▸) from the Grant laboratory, *WEBnm@* from the Reuter laboratory (Tiwari *et al.*, 2014 ▸), *ENCORE* from the Lindorff-Larsen laboratory (Tiberti *et al.*, 2015 ▸), *MODE-TASK* (Ross *et al.*, 2018 ▸), *MD-TASK* (Brown *et al.*, 2017 ▸) and *MDM-TASK-web* (Sheik Amamuddy *et al.*, 2021 ▸) from the Atilgan and Tastan Bishop laboratories and *MAVENs* (Zimmermann *et al.*, 2011 ▸) from the Jernigan laboratory. A similar trend towards more flexible automation and pipelines has been seen for software more closely related to cryoEM analysis including *CCP-EM* (Burnley *et al.*, 2017 ▸), *RELION* (Zivanov *et al.*, 2018 ▸), and *Xmipp* (Strelak *et al.*, 2021 ▸) and *Scipion* (Jimenez-Moreno *et al.*, 2021 ▸) from the Carazo laboratory.

A number of these packages have grown in similar directions in recent years, with a major feature being enriched ensemble analysis tools to perform more complicated comparisons of the conformational states and dynamics of large numbers of related structures (Mikulska-Ruminska *et al.*, 2019 ▸; Tiwari *et al.*, 2014 ▸; Tiwari & Reuter, 2018 ▸; Yao *et al.*, 2016 ▸; Zhang *et al.*, 2019 ▸). There has also been a great deal of focus in recent times on linking these techniques to lower resolutions with pseudoatoms (Chen & Ludtke, 2021 ▸; Jonić & Sorzano, 2016*a* ▸; Kawabata, 2018 ▸; Zhang, Krieger, Mikulska-Ruminska *et al.*, 2021 ▸). We review these developments with a focus on examples from our recent work, including version 2.0 of the *ProDy* API (Zhang, Krieger, Zhang *et al.*, 2021 ▸).

## 2. Computational biophysics methods for different timescales: a case of horses for courses?

**Figure 1:**
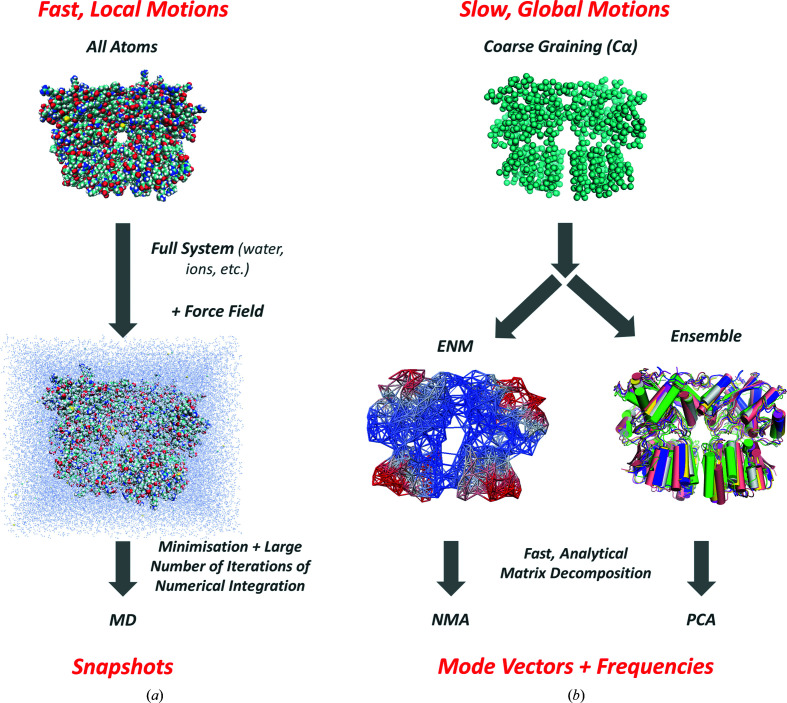
Different methods and representations for different scales of motion. (*a*) Local motions require specialized force fields, extensive energy minimization and many iterations of molecular dynamics (MD) simulations to capture the effects of detailed interactions between atoms, including surrounding waters and ions. (*b*) Global motions can be calculated with coarse-grained (CG) representations such as one node per residue at the C^α^ atom, which can be used with elastic network models (ENMs) and conformational ensembles for normal mode analysis (NMA) and principal component analysis (PCA). These approaches provide fast, analytical methods for extracting mode vectors via matrix decomposition. The structure illustrated is a GluA3 glutamate receptor N-terminal domain dimer, which we have studied extensively using all of these methods (Krieger *et al.*, 2015 ▸; Lee *et al.*, 2019 ▸).

Protein dynamics can take place on a range of length scales and timescales from vibrations of individual chemical bonds on a sub-ångström length scale and the femtosecond timescale to global reconfigurations of domains and subunits spanning tens to hundreds of ångströms on a microsecond-to-millisecond timescale. Accordingly, different methods and representations are appropriate for studying protein dynamics in line with these different scales (Fig. 1 ▸).

At the local scale lies the most popular of all molecular biophysics methods: all-atom MD simulations (Hollingsworth & Dror, 2018 ▸). MD simulations use detailed force fields to calculate all of the atom interactions within a system, including those involving both the protein or complex under study and the surrounding water and ions (Fig. 1 ▸ *a*), as well as the lipid bilayer for membrane proteins. They then numerically solve Newton’s equations of motion over a large number (10^7^–10^10^) of time steps (of 1–2 fs), allowing movements to be followed in full-atomic detail, but at great computational cost. This large number of time steps can be very valuable in certain situations, such as for drug design (Śledź & Caflisch, 2018 ▸; Yu & MacKerell, 2017 ▸) and in investigations of mutation effects, when specific atom interactions may be important. However, dedicated supercomputers (such as Anton; Shaw *et al.* 2009 ▸, 2014 ▸) or sophisticated enhanced sampling algorithms (Abrams & Bussi, 2014 ▸; Bernardi *et al.*, 2015 ▸; Harpole & Delemotte, 2018 ▸; Zuckerman & Chong, 2017 ▸; Pietrucci, 2017 ▸) are required to simulate the cooperative motions of large macromolecular assemblies that take place on timescales longer than a few hundred nanoseconds, and other approaches may be preferable, if not required, to accurately capture such global motions.

By nature, global dynamics involve large, concerted movements of a large part of the structure in which many atoms move together. These motions therefore do not require full-atomic descriptions and are suitably described at a more CG level. For example, one can model them at the amino-acid residue level and place a representative node based on the α carbon (C^α^; Fig. 1 ▸ *b*) or the average of all atoms belonging to that residue. As such, these models are amenable to faster analytical evaluation of conformational variabilities or fluctuations using PCA (often applied to a series of conformers of the same protein from experiments or simulations) or NMA (applied to a single representative structure), using linear algebra methods, as will be outlined in the next two sub­sections.

These methods also have important limitations. By their very nature, global motions lack atomic detail and can result in unrealistic deformations of bond lengths and angles. A number of hybrid methods that combine global dynamics methods with MD simulations have been developed to correct for unphysical deformations, provide conformers at atomic resolution and sample conformational landscapes and transitions, as discussed in our recent review (Krieger *et al.*, 2020 ▸). NMA, especially when used with ENMs, also has the dis­advantage that the calculated dynamics are constrained by the interactions found in the initial conformation and often fail to capture the rupture of domain or subunit interfaces, and may not perform well when starting from closed/compact conformations. More adaptive approaches such as MD, where the interactions and forces are recalculated after every small change in structure, are sometimes better able to capture this, although this often requires lengthy or complicated simulations including enhanced sampling schemes such as steered MD or umbrella sampling (Lau, 2019 ▸; Pietrucci, 2017 ▸).

### 2.1. Essential dynamics of structural ensembles: global motions from related structures

It is often useful to describe the space of conformations with the help of 3*N*-dimensional vectors,

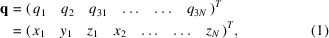

giving the 3D Cartesian positions (*x_n_*, *y_n_*, *z_n_*) of the *N* nodes (1 ≤ *n* ≤ *N*) of the structure. The fluctuations or displacements Δ**q** in these coordinates with respect to the equilibrium (or reference) coordinates are in turn described by the 3*N*-dimensional deformation vector

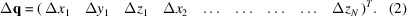

**Figure 2:**
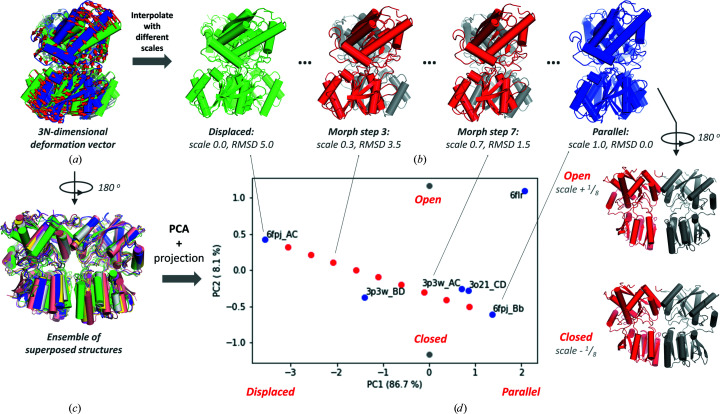
Global motions from structural comparisons illustrated for the GluA3 glutamate receptor N-terminal domain dimer. (*a*, *b*) Comparison of two structures by calculating a deformation vector between corresponding atom positions (*a*) and a morph (*b*). A view from one perspective shows an inter-subunit counter-rotation, resulting in a transition from displaced to parallel lower lobes. (*c*, *d*) Ensemble analysis using multiple structures (*c*) and PCA (*d*). A projection onto the subspace of the first two PCs (*d*) (left) allows a mapping of the conformational space of the structural ensemble in (*c*) (blue points labelled with PDB and chain IDs corresponding to the respective dimers) as well as the conformations from the morph in (*b*) (red points); the values along the axes show the r.m.s.d. contributed by PC1 and PC2 from the average at the origin. PC1 (*x* axis of the projection) accounts for most of the variation between the red points, supporting its correspondence to the displaced → parallel transition in (*b*), in line with PC1 having a directional overlap (correlation cosine) of 0.98 to the deformation vector. By contrast, PC2 (*y* axis) features an opening and closing motion of the lower lobes. This motion can be visualized by adding PC2 to the average conformer (in this case with 1/8 of its variance) in the positive and negative directions, generating two new structures, which are marked by grey points on the plot and illustrated on the right. The structures in (*c*) and (*d*) are rotated about the dimer interface relative to those in (*a*) and (*b*) as indicated by the rotation arrows.

The simplest example of this is a morph between two structures. Subtraction of the two coordinate sets after superposition gives the deformation vector needed to move the nodes from their positions in one structure to their positions in the other (Fig. 2 ▸ *a*). We can then visualize the motion associated with this vector by generating conformations along it using different scaling factors (Fig. 2 ▸ *b*). However, such morphing between two end points may give rise to unphysical conformers at the atomic level (for example, interpolation between two rotational isomeric states for C^α^—C^β^ bonds, *i.e.* *trans* and *gauche* ^±^ states separated by 120°, yields an unreal­istic high-energy state). We can refine this approach to investigate how a protein moves by analysing an ensemble of structures of that protein (Fig. 2 ▸ *c*), which can come from any source, including simulations and experiments, or by using physically plausible deformations for the structural components using, for example, the normal modes of motion. Homologous proteins may also be included to compare how their structures are related. A projection to a subspace of major changes in conformation also allows a clearer visual­ization of the dominant mechanisms of structural change that are usually insensitive to atomic-scale approximations.

The most widely used technique of this type of dimensionality-reduction approach is called essential dynamics analysis (EDA), which was first pioneered with MD simulations (Amadei *et al.*, 1993 ▸; García, 1992 ▸) and allows one to reduce the structural variation into a small set of essential ‘modes’ of motion and to create a low-dimensional mapping of the conformational landscape. Although other methods such as multi-dimensional scaling have also been used, we focus on the typical approach with PCA (Kitao & Go, 1999 ▸), which was shown to be useful in describing global protein dynamics from experimental and simulation ensembles in the 1990s (Amadei *et al.*, 1993 ▸; García, 1992 ▸; van Aalten *et al.*, 1997 ▸) and continues to be widely used to this day, including in our recent work (Zhang, Krieger, Mikulska-Ruminska *et al.*, 2021 ▸; Yang, Eyal *et al.*, 2009 ▸). The outputs are very easy to analyse and use, as we show below, enabling them to enhance sampling in simulations (Amadei *et al.*, 1996 ▸; Lange & Grubmüller, 2006 ▸) and the refinement of ensembles against X-ray crystallographic data (Romo *et al.*, 1995 ▸).

The main idea behind PCA is to decompose the structural variation into vector components and select the principal components that contribute the highest fractional variance, which tend to be global motions. The remaining components usually describe local rearrangements, which may not be so meaningful given the small data-set size and are usually ignored. This structural variation is described by the 3*N* × 3*N* positional covariance matrix **C**, the *ij*th element of which is the average of the dot products of the deviations Δ**q** of the *i*th and *j*th components of **q** in each conformation *M* from the average structure. An eigendecomposition of this matrix gives rise to a set of 3*N* eigenvectors **p**_*k*_ (or 3*N* − 6 nonzero eigenvectors, omitting those associated with the rigid-body deformations) with associated eigenvalues σ_*k*_ describing the directions of motion and their variance contributions, respectively:

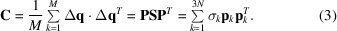




Each eigenvector **p**_*k*_ is 3*N*-dimensional, giving a relative extent of motion of each of the *N* nodes away from the average structure in the 3D Cartesian coordinates. Their variance contribution gradually decreases and the first two to five nonzero eigenvectors are usually considered principal components (PCs).

One can add one of these vectors or any linear combination of them to the average structure or any other conformer to generate a new conformation and thereby visualize the associated motions as above (Fig. 2 ▸ *d*, right). The scaling factors along each of the PCs can be used to define a new low-dimensional space spanned by the orthonormal PCs. The structures in the ensemble can be projected onto this space by taking the dot products of the deviations and the mode vectors, yielding a set of scaling factors for each structure. This structure mapping gives an idea of the conformational space, *i.e.* how the different structures in the ensemble are related to each other (Fig. 2 ▸ *d*, left).

If the ensemble is large (and unbiased) enough, it is also possible to calculate the occupancy of different regions in this space using binning or kernel density estimators to obtain a first estimate of the conformational energy landscape. This analysis has been performed for microsecond simulations of the small protein BPTI using the Anton supercomputer (Gur *et al.*, 2013 ▸) and for the dopamine transporter (Cheng *et al.*, 2018 ▸), allowing the identification of interconverting substates (clustering in the PC space) and the corresponding well depths and barriers in the free-energy landscape.

### 2.2. NMA and ENMs: an old partnership with continuing success

Normal mode analysis (NMA) calculates modes of motion from single structures. It is based on a Taylor expansion of the interatomic interaction potential *V* around a given conformation **q** ^0^,

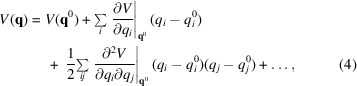

where **q** is the coordinate vector for any conformation (equation 1) near **q** ^0^. When **q** ^0^ is at an energy minimum, we can treat the first two terms as zero. For the potential energy itself (first term), this requires shifting all of the values of the potential so that the minimum is zero, and the slope of the potential energy landscape (the second term) is also zero at the minimum, by definition. Therefore, the third (second derivative) term dominates as the remaining terms are negligible, reducing the potential to a quadratic approximation, 

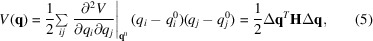

where **H** is the Hessian matrix of second derivatives, which is the inverse of the fluctuation covariance matrix (Bahar *et al.*, 2010 ▸, 2017 ▸). It can be shown that solving the equations of motion is equivalent to solving an eigenvalue problem (Bahar *et al.*, 2010 ▸, 2017 ▸), giving rise to a set of oscillatory motions around the energy minimum. The eigenvalue decomposition of the Hessian yields the (3*N* − 6) nonzero normal modes. The eigenvectors describe the directions of collective motions in each mode, and the corresponding eigenvalues are the squared frequencies of these motions. Note that the first six modes correspond to the rigid-body movements associated with the three translational and three rotational degrees of freedom of the system and have zero eigenvalues, which leads to nonzero mode 1 sometimes being called mode 7.

**Figure 3:**
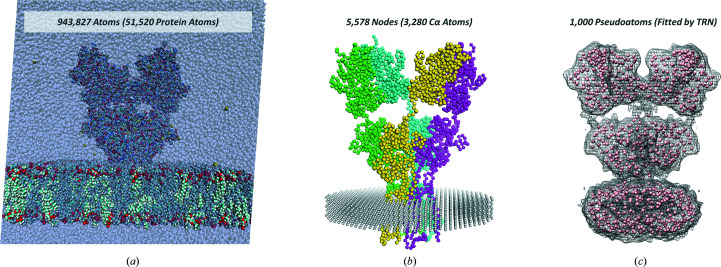
Comparison of different representations for a tetrameric membrane protein resolved by cryoEM. A GluA2 glutamate receptor (EMDB entry EMD-2680 and PDB entry 4uqj; Meyerson *et al.*, 2014 ▸) is shown as part of a full simulation system with explicit waters, ions and lipid molecules (membrane) (*a*), as an anisotropic network model (ANM) embedded in a membrane lattice that is also treated as an ANM (*b*) and as a set of pseudoatoms fitted using the TRN algorithm for vector quantization (*c*).

Traditionally, NMA would be performed using full-atomic MD force fields (Fig. 1 ▸ *a*), which requires extensive energy minimization in implicit solvent or explicit water molecules and ions beforehand to ensure that the system is at an energy minimum. This process would significantly slow down the calculation overall. Around the turn of the century, simpler potentials called ENMs were invented, which allow much more efficient NMA. The applicability of harmonic potentials to robustly evaluate the global modes was first demonstrated by Monique Tirion, who applied a harmonic potential to all atomic interactions with a uniform force constant and a single cutoff distance (Tirion, 1996 ▸). Any pairs of atoms with a distance shorter than or equal to this cutoff distance were treated as beads connected by springs, and any atoms at a longer distance were considered not to interact. This pioneering study led to the introduction of the first elastic network model, the Gaussian network model (Bahar *et al.*, 1997 ▸), and analytical evaluation of normal modes, followed by the widely used anisotropic network model (ANM), which introduces a level of coarse graining of one node per residue (at the locations of the C^α^ atoms; Fig. 1 ▸ *b*; Atilgan *et al.*, 2001 ▸; Eyal *et al.*, 2006 ▸; Tama & Sanejouand, 2001 ▸) or even higher (Doruker *et al.*, 2002 ▸). Other elastic network models also exist with different distance dependencies (Hinsen, 1998 ▸; Yang, Song *et al.*, 2009 ▸) as well as alternative methods of coarse graining including vibrational subsystem analysis (VSA; Hinsen *et al.*, 2000 ▸; Ming & Wall, 2005 ▸; Woodcock *et al.*, 2008 ▸; Zheng & Brooks, 2005 ▸; Zhang, Zhang *et al.*, 2021 ▸), rotation and translation of blocks (RTB; Durand *et al.*, 1994 ▸; Schuyler & Chirikjian, 2004 ▸, 2005 ▸; Tama *et al.*, 2000 ▸) and Markovian hierarchical coarse graining (Chennubhotla & Bahar, 2007*a* ▸). These models have been key to the popularization of NMA by making it much more tractable on laptops as well as dedicated webservers (Camps *et al.*, 2009 ▸; Eyal *et al.*, 2015 ▸; Krüger *et al.*, 2012 ▸; Li *et al.*, 2017 ▸; Lindahl *et al.*, 2006 ▸; López-Blanco *et al.*, 2014 ▸; Tiwari *et al.*, 2014 ▸). ENMs have also been developed for nucleic acids (Zimmermann & Jernigan, 2014 ▸; Bahar & Jernigan, 1998 ▸) and lipids surrounding membrane proteins (Lezon & Bahar, 2012 ▸; Zhang, Zhang *et al.*, 2021 ▸) (see Fig. 3 ▸).

A key feature of ENMs is that they treat the known structure as an energy minimum (a reasonable assumption as it has been observed experimentally) and allow the direct use of an analytical expression for the Hessian. In the ANM for example, **H** is a 3*N* × 3*N* matrix (for a system of *N* residues), the 3 × 3 super-elements of which are simply

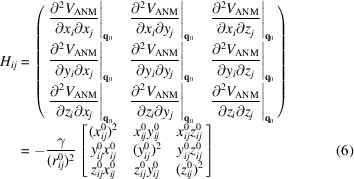

for *i* ≠ *j* if 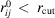 and zero otherwise. The diagonal super-elements of **H** are 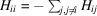 .

Here, γ is the uniform force constant used for all pairs within a distance of *r*_cut_, 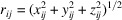 is the instantaneous distance between nodes *i* and *j* (where *x_ij_* ≡ *x_j_* − *x_i_* and the superscript 0 refers to the equilibrium (or experimentally resolved) structure. The ANM potential is defined as 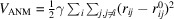 , where the summation is over all pairs with 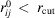 . Use of equation (6) significantly simplifies the evaluation of normal modes (upon its eigenvalue decomposition) without the need to perform simulations or energy minimization and without compromising the accuracy of the global modes.

## 3. Revisiting ensemble analysis: large-scale, high-throughput comparisons of structure, dynamics and evolution

The wealth of structures that are now available has required us to create and employ a more efficient high-throughout approach, which has in turn enabled us to perform unprecedented large-scale analyses. The developments in this area fall into two classes: ensemble construction and high-throughput comparative NMA for characterizing the *signature dynamics* of protein families, which are outlined in the two subsections below. A larger number of structures covering more of the conformational space allows the calculation of more relevant global modes of motion and better approximation of populations and energy landscapes, but also poses challenges for the construction of high-quality ensembles. As described in Section 2.1, this is critical for PCA as calculating global dynamics depends on accurately defining the average structure and the deviations from it. Likewise, comparative approaches require that equivalent parts of structures are indeed being compared, and ensembles are also a good tool for this.

### 3.1. New structure collection and alignment methods for ensembles

The starting point for any ensemble analysis is a collection of structures that have been optimally aligned and superposed. This can be performed in a number of ways depending on the problem at hand and the data that are available. The major source of structures is the continually growing Protein Data Bank (PDB; Berman *et al.*, 2000 ▸), which is celebrating its 50th anniversary (Berman & Gierasch, 2021 ▸) and now includes ∼175 000 entries (Velankar *et al.*, 2021 ▸). Structures can be downloaded directly from the PDB via one of their websites, or programmatically via their FTP or HTTP resources as is performed by *ProDy* (Bakan *et al.*, 2011 ▸, 2014 ▸; Zhang, Krieger, Zhang *et al.*, 2021 ▸). The PDB web tools and APIs are very advanced and support a wide range of queries using PDB IDs, sequences, clusters with particular sequence identities, and IDs from other databases such as UniProt (UniProt Consortium, 2021 ▸). There are also a number of web servers that can perform sequence- and structure-based searches against the PDB, including NCBI *BLAST* (Johnson *et al.*, 2008 ▸; Boratyn *et al.*, 2013 ▸; Altschul *et al.*, 1990 ▸), *HMMER* (Eddy, 2011 ▸; Finn *et al.*, 2011 ▸) and *DALI* (Holm & Laakso, 2016 ▸), as well as protein-family databases such as Pfam (Mistry *et al.*, 2021 ▸), InterPro (Blum *et al.*, 2021 ▸) and CATH (Sillitoe *et al.*, 2021 ▸). Interfaces for many of these tools have been added to *ProDy* (Zhang, Krieger, Zhang *et al.*, 2021 ▸; Zhang *et al.*, 2019 ▸) and *Bio*3*D* (Grant *et al.*, 2021 ▸).

There are many methods for aligning proteins based on their sequence (Altschul & Pop, 2017 ▸), structure (Ma & Wang, 2014 ▸) and even dynamics (Micheletti, 2013 ▸), which may be applicable depending on the situation. Sequence alignments are usually good enough unless there is very poor sequence similarity. Structure is more conserved than sequence and can therefore work well for finding alignments, but may come at further computational expense and thus is not advised when sequence-based methods suffice. In our experience with *ProDy*, we have generally found that the pairwise sequence-alignment methods implemented in *Biopython* (Cock *et al.*, 2009 ▸) work well in many cases and that *DALI* pairwise structural alignment (Holm & Laakso, 2016 ▸) works well in many others (Zhang *et al.*, 2019 ▸).

One efficient method for alignment and superposition is to perform pairwise calculations, comparing all sequences/structures with an initial reference. A first multiple sequence alignment and aligned structural ensemble can then be created based on this and manually curated, with some refinement being applied manually or using multiple sequence-alignment tools where necessary. Finally, these alignments are used to iteratively superpose the structures until the average converges. Some trimming of flexible termini and loops may also be performed to avoid their nuisance contributions, which are often referred to as ‘tip effects’ (Lu *et al.*, 2006 ▸; Woodcock *et al.*, 2008 ▸).

### 3.2. Comparative NMA reveals signature dynamics and specialization

Early in the development of ENMs, it was observed that similar protein structures had similar global dynamics (Keskin *et al.*, 2000 ▸; van Vlijmen & Karplus, 1999 ▸). With this came a realization that one could learn about the function of a protein by comparing its dynamics with those of related proteins. It was also realized that different conformations of the same protein may have considerable differences in dynamics and that evaluating ensemble averages may give a better description of the overall dynamics of proteins (Batista *et al.*, 2010 ▸; van Vlijmen & Karplus, 1999 ▸). With the growing wealth of structures that are available, it became possible to more systematically address questions about the relationships between sequence, structure, dynamics, function and evolution (Fuglebakk *et al.*, 2015 ▸; Liberles *et al.*, 2012 ▸; Liu & Bahar, 2012 ▸). This led various computational biophysics groups to come up with pipelines for performing NMA on ensembles of related structures and comparing the results, including our *SignDy* pipeline for signature dynamics (Mikulska-Ruminska *et al.*, 2019 ▸; Zhang *et al.*, 2019 ▸) within *ProDy*, and similar pipelines in *WEBnm@* (Tiwari *et al.*, 2014 ▸) and in *Bio*3*D* (Skjaerven *et al.*, 2014 ▸) and *Bio*3*D-Web* (Jariwala *et al.*, 2017 ▸).

Preliminary studies, including comparisons of smaller sets (Dutta *et al.*, 2015 ▸; Fuglebakk *et al.*, 2012 ▸; Krieger *et al.*, 2015 ▸; Liu & Bahar, 2012 ▸; Maguid *et al.*, 2005 ▸; Ponzoni *et al.*, 2018 ▸), and reviews of available methods (Fuglebakk *et al.*, 2015 ▸; Haliloglu & Bahar, 2015 ▸; Micheletti, 2013 ▸) were key in defining important steps of the pipelines. These included which measures and comparisons to calculate, how to handle positions with insertions and deletions in some proteins and how to match similar modes. For example, it was found that root-mean-square fluctuations (RMSFs) or mean-square fluctuations (MSFs) did not provide sufficient information by themselves and covariance matrices should also be used, and the covariance overlap developed by Berk Hess (Hess, 2002 ▸) was found to be a very good measure of dynamics similarity over sets of modes (Fuglebakk *et al.*, 2012 ▸, 2015 ▸). We also confirmed that VSA was a good way to handle the tip effect from loops and other insertions (Dutta *et al.*, 2015 ▸; Woodcock *et al.*, 2008 ▸). Once these issues had been addressed, it was possible to perform much larger-scale analyses including large superfamilies such as enzymes with the triosephosphate isomerase (TIM) barrel fold (Tiwari & Reuter, 2016 ▸; Zhang *et al.*, 2019 ▸) as well as a systematic analysis of the conservation of different dynamic regions across a large data set of CATH families (Zhang *et al.*, 2019 ▸).

We discovered that there are indeed conserved signature dynamics that show evolutionary patterns dependent on how global/collective they are (Zhang *et al.*, 2019 ▸). The lowest-frequency, most global modes were unsurprisingly the most conserved, as expected from previous studies, but we were also able to observe the conservation of high-frequency, local modes in line with their proposed roles in structural stability. In between, there were many moderately conserved but fairly global modes in what we termed the low-to-intermediate frequency regime, which appeared to drive subfamily specification (Zhang *et al.*, 2019 ▸). We also showed that it was possible to classify structures based on their dynamics and construct phylogenetic trees, similar to as can be performed with sequences and structures (Zhang *et al.*, 2019 ▸).

## 4. Further coarse-graining: a number of different lower resolution representations of use in different pipelines

For the large structures being resolved by cryoEM, MD simulations are prohibitively expensive computationally, especially for membrane proteins, where the membrane should also be included (Fig. 3 ▸ *a*), and it is even challenging to use residue-resolution representations for PCA and NMA (Fig. 3 ▸ *b*). The dynamics of the system may also lead to lower resolution maps where alternative representations may be more useful. A number of such low-resolution representations of groups of atoms have been developed, which can loosely be referred to as pseudoatoms. These representations are all based on fitting roughly spherical objects into the density maps, which can still be treated in a similar fashion to atoms (Fig. 3 ▸ *c*). Other approaches have also been employed, including fluctuating finite element analysis (FFEA; Solernou *et al.*, 2018 ▸), which fits tetrahedral elements into the density map using meshing tools and applies its own physical model based on an extension of finite-element analysis from engineering to include thermal fluctuations.

Three main types of methods exist for pseudoatom fitting. The first is vector quantization (VQ), where the cryoEM map is divided into regions whose centres are defined by codebook vectors. The most widely used version of this is a machine-learning method called the topology-representing network (TRN) or neural gas network, developed by Klaus Schulten’s group (Martinetz & Schulten, 1994 ▸; Wriggers *et al.*, 1998 ▸). TRN was used extensively at the turn of the century by Wriggers and coworkers, who created the *Situs* package that uses TRN-based VQ for docking proteins/domains into cryoEM maps as well as flexible fitting optimizations (Wriggers, 2010 ▸; Wriggers *et al.*, 1999 ▸), exploration of global modes (Chacón *et al.*, 2003 ▸; Tama *et al.*, 2002 ▸) and the development of an ENM for even coarser-grained cases (Stember & Wriggers, 2009 ▸). Independently, the Ma laboratory showed many successful applications of this technique, which they called the quantized elastic deformational model (Beuron *et al.*, 2003 ▸; Kong *et al.*, 2003 ▸; Ming, Kong, Lambert *et al.*, 2002 ▸; Ming, Kong, Wakil *et al.*, 2002 ▸). More recently, this technique has been used in the *gamma-TEMPy* method for assembly fitting of subunits into cryoEM maps (Pandurangan *et al.*, 2015 ▸).

Given its success in all of these applications but the difficulty in integrating it with other protein dynamics analyses, we recently implemented it into *ProDy* in the context of the *CryoDy* pipeline for dynamics from cryoEM (Zhang, Krieger, Mikulska-Ruminska *et al.*, 2021 ▸). We applied and tested it on the mammalian chaperonin TRiC/CCT, demonstrating its utility for NMA, Markovian hitting time analysis of allosteric signal flow (Chennubhotla & Bahar, 2007*b* ▸) and PCA. This pipeline includes not only TRN and its connection to ENM NMA, but also a first nearest-neighbour mapping between pseudoatoms and atoms and a dynamics-based clustering scheme for domain/subunit identification (Zhang, Krieger, Mikulska-Ruminska *et al.*, 2021 ▸). We also implemented the adaptive ANM method for NMA-guided transition sampling (Yang, Majek *et al.*, 2009 ▸) within *ProDy* as part of this pipeline, allowing this method to be used with pseudoatoms.

An alternative method is to fit spherical Gaussians of fixed standard deviation using an approximation-accuracy control algorithm (Jonić & Sorzano, 2016*a* ▸,*b* ▸). This method has found utility in a number of applications including the denoising of cryoEM maps (Jonić *et al.*, 2016 ▸) and NMA (together with Florence Tama; Nogales-Cadenas *et al.*, 2013 ▸), which was useful for continuous flexibility analysis in *Hybrid Electron Microscopy Normal Mode Analysis* (*HEMNMA*; Jin *et al.*, 2014 ▸; Sorzano *et al.*, 2014 ▸) and the cryoEM map comparison tool *StructMap* (Sanchez Sorzano *et al.*, 2016 ▸). These two methods are implemented in the *ContinuousFlex* plugin of *Scipion* 3.0 (Harastani *et al.*, 2020 ▸) and continue to be developed further, including in the recent *HEMNMA-*3*D* method for subtomograms from cryo-electron tomography (Harastani *et al.*, 2021 ▸).

The last approach for pseudoatom fitting is to use a Gaussian mixture model (GMM), as pioneered by Takeshi Kawabata, who also used it for fitting (Kawabata, 2008 ▸). This has the benefit that it can also easily be used to represent atomic models, allowing it to form the basis of the *Omokage* server for shape-similarity searches against the PDB and EMDB (Suzuki *et al.*, 2016 ▸) and in integrative modelling (Bonomi *et al.*, 2019 ▸) as well as ensemble flexible fitting with the *EM metainference* (*EMMI*) algorithm (Bonomi *et al.*, 2018 ▸). In theory TRN can also be applied to atomic models, but not as easily. Together with a new, more efficient GMM fitting program (Kawabata, 2018 ▸) and a very recent implementation within *EMAN*2 that has been used for continuous heterogeneity analysis (Chen & Ludtke, 2021 ▸), this is clearly a fast-moving area.

## 5. Conclusion

Elastic network models continue to provide very useful CG representations for the efficient analyses of global dynamics of biomolecular complexes. They permit the extraction of global mode vectors from matrix-decomposition methods, such as NMA of individual structures and PCA of structural ensembles, which are robust to resolution. Recent innovations in computational biophysics enable improved ensemble analyses, including comparative NMA as well as pseudoatom fitting approaches, permitting the analysis of larger ensembles and increasingly larger, more dynamic complexes. A trend towards customisable pipelines, such as *SignDy* and *CryoDy*, makes these techniques even more widely usable and we expect great developments in the future, aided by continuing developments in structural biology including the availability of structural models resolved by *AlphaFold*2 (Jumper *et al.*, 2021 ▸; Varadi *et al.*, 2021 ▸). The next big area is clearly continuous heterogeneity/dynamics analysis of cryoEM images (Chen & Ludtke, 2021 ▸; Giraldo-Barreto *et al.*, 2021 ▸; Herreros *et al.*, 2021 ▸; Sorzano *et al.*, 2019 ▸) in place of the existing discrete classification approaches, which could benefit from a better connection to such computational biophysics approaches.
